# Locally-Sourced Animal Protein Hydrolysates in High-Plant-Protein Diets Can Promote European Seabass Growth and Nutrient Utilization, Reducing Reliance on Fishmeal

**DOI:** 10.1155/anu/3415083

**Published:** 2025-11-18

**Authors:** Luciano Rodrigues-dos-Santos, Ana Basto, Marta Monteiro, Carla António, Ana M. Rodrigues, Tiago Sá, Cristina Velasco, Rui Martins, Ana Rosa, Manuela Pintado, André Almeida, Luisa M. P. Valente

**Affiliations:** ^1^CIIMAR/CIMAR LA, Interdisciplinary Centre of Marine and Environmental Research, University of Porto, Av. General Norton de Matos S/N, Matosinhos 4450–208, Portugal; ^2^ICBAS, School of Medicine and Biomedical Sciences, University of Porto, Rua de Jorge Viterbo Ferreira 228, Porto 4050–313, Portugal; ^3^ETSA, I.T.S., Industry of By-Products, S.A. (ETSA Group), Loures 2660–119, Portugal; ^4^SEBOL, Commerce and Industry of Tallow, S.A. (ETSA Group), Loures 2660–119, Portugal; ^5^CBQF, Centre for Biotechnology and Fine Chemistry, School of Biotechnology, Catholic University of Portugal, Rua Diogo Botelho 1327, Porto 4169–005, Portugal; ^6^Plant Metabolomics Lab Portugal, Tapada da Ajuda, Lisboa 1349–017, Portugal; ^7^Associate Laboratory TERRA, Forest Research Centre, School of Agriculture, University of Lisbon, Tapada da Ajuda, Lisboa 1349–017, Portugal; ^8^iBET, Institute of Experimental and Technological Biology, Apartado 12, Oeiras 2781–901, Portugal

**Keywords:** circular bioeconomy, Fe-bioavailability, muscle metabolome, plant-based aquafeeds, protein hydrolysates

## Abstract

Upcycling meat and fish by-products into bioactive protein hydrolysates promotes zero-waste practices within the circular bioeconomy and provides locally-sourced alternatives to replace fishmeal (FM) in aquafeeds. In this study, novel hydrolysates were developed from blue shark skin, fish by-products, and swine processed animal protein, aiming to reduce the high-quality FM inclusion in European seabass (*Dicentrarchus labrax*) diets. Four isoproteic (53%) and isolipidic (18%) plant-based diets were formulated: a control (CTRL) diet containing 12.5% FM was compared to three experimental diets, including 3% of each hydrolysate at the expense of FM-protein: SHARK, FISH, and SWINE. Seabass juveniles (13 g) stocked in 160 L tanks (3.9 kg/m^3^) were fed the diets in triplicate, three times daily until satiety, for 89 days. All diets were equally well accepted, promoting similar feed intake, daily growth index (1.6), and efficient feed conversion ratio (1.0–1.1). All fish at least quadrupled their initial size, but those fed the SWINE diet showed the highest body weight (55 g) and significantly higher condition factor (1.22), hepatosomatic index (HSI) (1.6), and viscerosomatic index (VSI) (8.4). Intestinal integrity remained similar across treatments, and apparent digestibility coefficients (ADCs) of protein and fat were above 96%. Iron (Fe) ADC was at least doubled with hydrolysates inclusion. Whole-body and muscle composition were similar across groups. However, the muscle metabolome of the SHARK-fed fish exhibited higher levels of glycolytic intermediates and lower levels of glucogenic amino acids (AAs) and fumarate, suggesting an increased catabolic activity. However, these changes were not reflected in fish growth performance or muscle flesh quality, which remained similar among treatments.

## 1. Introduction

The agri-food sector significantly contributes to global waste, with ~33% of all food produced being lost or wasted throughout the supply chain [1]. Among the largest contributors are the meat and fish processing industries, which generate substantial quantities of protein-rich by-products, such as heads, bones, carcasses, blood, skin, viscera, hooves, and feathers [2]. Currently, this sector is increasingly adopting circular economy approaches to repurpose these low-value products, addressing the European zero-waste strategy [3] and the United Nations Sustainable Development Goal 12 – Responsible Production and Consumption (SDG 12). One promising strategy to maximize resource efficiency is the production of protein hydrolysates from these by-products. Through hydrolysis, proteins are broken down into highly digestible peptides and free amino acids (AAs), which can then be utilized as high-value ingredients in animal feeds. Many of these peptides, rich in essential AAs (EAAs), offer physiological benefits to livestock and aquaculture species, beyond their nutritional value [4]. Supplementing feed with certain hydrolysates has been shown to boost animal growth and welfare, while contributing to more efficient and sustainable animal farming practices [5]. Nevertheless, evidence indicates that variations in raw materials, hydrolysis and processing conditions, purification methods, and administration doses can significantly alter the outcomes across different species [2].

Protein hydrolysates derived from fish by-products have been widely used in several fish species and have been shown to promote weight gain in red seabream (*Pagrus major*), barramundi (*Lates calcarifer*), and European seabass (*Dicentrarchus labrax*) [6–11]. These effects have been attributed to their high palatability [12–14], a balanced AA profile [5], high digestibility [15], and enhanced intestinal absorption [13]. Fish hydrolysates (FISH_Hs) have also been reported to modulate energetic metabolism in liver and muscle tissues in turbot (*Scophthalmus* maximus) [16, 17], as well as muscle textural properties and myofiber density in crucian carp (*Carassius auratus*) [18]. Likewise, hydrolysates from animal by-products, such as those from swine, have been shown to improve fish growth performance [19], enhance protein digestibility [20], increase survival rate against pathogenic infections [21], and bolster the response to oxidative stress [22] in economically relevant species, including European seabass, gilthead seabream (*Sparus aurata*) and Nile tilapia (*Oreochromis niloticus*). Such physiological responses may be associated with the presence of bioactive peptides, defined as short sequences of 2–50 AAs [23] capable of interacting with biological processes and eliciting functional responses at the cellular or systemic level [24]. The evidence indicates that, beyond their nutritional contribution, such hydrolysates may also exert bioactive functions influencing fish metabolism, immunity, and stress resistance in fish [25].

Given the fast expansion of the aquaculture industry and growing sustainability concerns, fishmeal (FM)—the ideal protein source for most carnivorous fish—must now be considered a strategic ingredient in fish diets due to its low availability. Plant proteins (PPs) have been extensively used as the main alternative sources [26], but high inclusion levels often lead to adverse effects in carnivorous fish [27]. The presence of unpalatable compounds and antinutritional factors (ANFs) in PPs hinders proper nutrition of these species, as it compromises normal feed intake and nutrient absorption, which are essential to meet the high energy and protein demands during the growth phase [27]. Moreover, the deficiency of lysine and methionine, commonly associated with PPs [28], poses a significant challenge, as these AAs play crucial physiological roles to ensure good fish health and fast muscle growth [29]. It is well documented that low FM plant-based diets for European seabass are frequently associated with reduced feed intake and nutrient digestion capacity, leading to impaired feed efficiency and growth performance, and compromised flesh quality traits [30–34]. The inclusion of hydrolysates with bioactive potential, in plant-based diets for carnivorous fish species, may effectively reduce dietary FM inclusion and mitigate the negative impacts associated with plant-based ingredients in aquafeeds [8, 11, 21].

This study hypothesizes that incorporating locally-sourced animal protein hydrolysates into high PP diets can promote fish growth and nutrient utilization, potentially reducing the reliance on FM in diets for European seabass juveniles. To test this hypothesis and unravel the underlying physiological mechanisms, we relied on an innovative, comprehensive approach merging classical and cutting-edge tools, like zootechnical data, histology, and metabolomics, to demonstrate the potential of a novel renewable source of bioactive compounds for aquafeeds.

## 2. Materials and Methods

### 2.1. Ethical Issues

All procedures and the experimental trial complied with European Union (EU) Directive 2010/63/EU and adhered to FELASA category C recommendations. All protocols were conducted in accordance with ethical standards, with prior approval from the Portuguese Veterinary Authority (1005/92, DGAV-Portugal) and CIIMAR Animal Welfare Body (ORBEA_CIIMAR_18_2017).

### 2.2. Hydrolysates

Three locally sourced animal by-products (i.e., from shark, fish, and swine), classified as Category 3 materials, were collected and processed by ETSA-SGPS, S.A. in compliance with Regulation (European Commission, EC) No 1069/2009 and its implementing Regulation (EC) No 142/2011, for the subsequent production of protein hydrolysates. The Shark hydrolysate (SHARK_H) was obtained from blue shark (*Prionace glauca*) skin, a by-product of fillet processing for human consumption, supplied by Brasmar -Trade Food S.A. (Guidões, Portugal). The FISH_H was produced from fish by-products (multispecies) obtained from various retailers and distributors in the national fish processing and canning industries. Both SHARK_H and FISH_H were produced by enzymatic hydrolysis with alcalase under previously optimized conditions [35]. The swine hydrolysate (SWINE_H) was generated through the hydrolysis of processed swine by-products, without chemical additives (Patent Application PCT/IB2024/061806). In all cases, the resulting protein fraction was concentrated by rotary evaporation and spray-dried. The production process and chemical characterization of these hydrolysates have been described in detail by Monteiro et al. [35].

All hydrolysates had high protein (>81% dry matter, DM) and low fat (<0.3% DM) contents. Protein solubility differed among hydrolysates, being lowest in SHARK_H (32.7% total crude protein [CP]), intermediate in FISH_H (40.3% total CP), and highest in SWINE_H (54.0% total CP). SHARK_H had the lowest EAAs (31% total AAs) and highest collagenic AAs (47% total AAs) contents, whereas FISH_H showed the opposite trend, with the highest EAA (44% total AAs) and lowest collagenic AA content (24% total AAs). Regarding peptide molecular weight distribution, SHARK_H and FISH_H were characterized by a predominance of 1–3 kDa peptides, with FISH_H also displaying a higher proportion of <1 kDa peptides. In contrast, SWINE_H showed a more balanced distribution across the different molecular weight ranges.

### 2.3. Experimental Diets

Four isoproteic (52.9% DM), isolipidic (17.8% DM), and isoenergetic (22.8 kJ g^−1^ DM) diets were formulated: a control (CTRL) plant-based diet, containing 12.5% of FM and 69% of vegetable-protein sources (providing 18% and 82% of the dietary protein content, respectively), and other three experimental diets obtained by adding 3% of SHARK_H, FISH_H, and SWINE_H (SHARK, FISH, and SWINE, respectively), at the expense of FM protein (Tables 1 and 2). Yttrium (Y) oxide was added to the experimental diets (0.02% DM) as an inert marker to assess feed digestibility. The experimental diets (2 mm of pellet size) were formulated based on European seabass nutrient requirements [36] and extruded by SPAROS Lda. (Olhão, Portugal). Diets proximate composition, AAs and fatty acids (FAs) profiles, and mineral composition are shown in Tables 2 and 3.

### 2.4. Growth Trial and Sampling Procedures

European seabass juveniles were obtained from a commercial fish farm (Acuinuga, S.L., Spain) and transported to the CIIMAR facilities (Matosinhos, Portugal). Fish were then subjected to a quarantine period of 15 days. In this period, fish were hand-fed a commercial diet (AQUASOJA, Portugal; 50% CP and 20% crude fat [CF] on DM basis). Before the onset of the trial, all fish were fasted for 24 h period, then slightly anesthetized (2-phenoxyethanol, 60 μL L^−1^), and individually weighed (12.6 ± 1.4 g) and measured (total length: 10.7 ± 0.5 cm) to establish homogeneous groups among the 12 tanks. Twelve groups of 50 European seabass (density: 3.9 kg m^−3^; coefficient variation: 11% within tanks) were then distributed across 160 L fiberglass tanks, within a recirculating aquaculture system (RAS). Each tank was supplied with seawater (33‰), mechanically filtered and oxygenated (>90% saturation), at a flow rate of 10.6 L min^−1^, and maintained at 22°C. Physical and chemical water parameters (temperature, salinity, oxygen saturation, redox potential, pH, and concentration of nitrogenous compounds) were monitored daily throughout the trial and maintained at optimal levels for European seabass [37]. After distribution among the tanks, 20 fish from the initial stock were euthanised by anesthetic overdose with 0.05% (v/v) 2-phenoxyethanol and stored at −20°C until analysis of the initial whole-body composition. The experimental diets were randomly allocated to triplicate tanks, and fish were fed three times daily to apparent satiation using automatic feeders for 89 days. The daily feed allowance was adjusted individually for each tank based on visual inspection of feed consumption. When all pellets were rapidly consumed, the following day's ration was increased by 5%. In contrast, if uneaten pellets were observed, the ration was reduced until no feed losses occurred. To ensure accurate calculation of feed intake, uneaten pellets were collected from each tank after every meal, freeze-dried, and weighed. By the end of the growth trial, all fish were individually weighed and measured to evaluate growth performance, after following the pre-weighing fasting and anesthesia protocol mentioned earlier. Ten fish per tank (30 fish per treatment) were sacrificed by spinal cord section, and the intestine with visceral fat and liver were collected and weighed to determine the viscerosomatic index and hepatosomatic index (VSI and HSI, respectively) indexes. One portion of dorsal muscle tissue (2.5–3.0 g) from five of those fish per tank was sampled, immediately frozen in liquid nitrogen (N), and stored at −80°C for subsequent nutritional composition evaluation and metabolomic analysis. Additionally, a section of the anterior region of the intestine (immediately after the pyloric ceca) was sampled, carefully washed, and fixed in 4% formaldehyde (pH 7.0) for histological analysis. The other five fish sacrificed from each tank were pooled and stored at −20°C for subsequent final whole-body composition determination.

### 2.5. Digestibility Trial

After the end of the growth trial, 48 fish remaining from each dietary treatment were divided into three homogeneous groups of 16 fish (53.0 ± 3.1 g) and transferred to a RAS with 50L fiberglass tanks (4 L min^−1^ flow rate) equipped with feces sedimentation columns (Guelph system), designed according to Cho and Slinger [38]. The fish were maintained under the same environmental conditions as during the growth trial and were fed the same experimental diets three times daily. Fish underwent a 15-day acclimation period to the new tanks, during which feed distribution was adjusted according to the tank with the lowest mean intake. After this period, feces were collected daily for 33 days from the sedimentation columns, prior to the distribution of the first meal. On each collection day, feces from each tank were centrifuged (3000 × *g*, 10 min, 4°C) to remove excess water and immediately stored at –20°C in containers specific to each tank. Daily collections from the same tank were pooled in the corresponding container to obtain composite samples for subsequent analyses.

After the last meal of the day, each tank was thoroughly cleaned to ensure the removal of any uneaten feed.

### 2.6. Chemical Analysis

#### 2.6.1. Proximate Composition of the Experimental Diets, Feces, and Fish Whole-Body and Muscle

Diets, freeze-dried whole-fish, dorsal muscle, and feces were ground, and feces were also sifted before chemical analysis, which was performed in duplicate and according to AOAC [39]. The DM and ash were determined by weight loss, after drying samples in an oven and combustion in a muffle furnace, respectively; CP was determined as total N by the Dumas method, using 6.25 as N-to-protein conversion factor [40]; CF was quantified after petroleum ether extraction in Soxhlet, and gross energy measured in an adiabatic bomb calorimeter as described by Monteiro et al. [41]. Phosphorus (P) content was assessed by colorimetric quantification of phosphates amount present in ash samples, as reported in Resende et al. [21]. In muscle samples, only DM and CP were assessed.

#### 2.6.2. Mineral Analysis

The mineral analysis (quantification of the inert marker [Y], aluminum [Al], calcium [Ca], cobalt [Co], copper [Cu], manganese [Mn], and iron [Fe] of diets and feces) was determined by inductively coupled plasma-mass spectrometry (ICP-MS), as described in Filipa-Silva et al. [42]. For analytical quality control purposes, three certified reference materials (CRMs) were used: Cabbage Powder (BCR-679), Hay Powder (BCR-129), and Aquatic Plant (BCR-670), all from the EU. The CRM was subjected to the same pre-treatment and analytical procedure of the samples.

#### 2.6.3. AA Analysis

The AA profile of diets was determined by ultra-performance liquid chromatography, after a sample hydrolysis with 6 M hydrochloric acid, during 48 h, at 116°C, as reported in Teodósio et al. [43].

#### 2.6.4. Total Lipids and FA Profile

Muscle total lipids were extracted and quantified according to the method described by Folch et al. [44], using Folch solution (dichloromethane: methanol 2:1 v/v containing 0.01% butylated hydroxytoluene, BHT). The FAs from experimental diets and muscle samples were derivatized by direct acid transmethylation according to Parrish et al. [45], with the modifications reported in Monteiro et al. [41]. The FA methyl esters thet resulted were identified and quantified by gas chromatography equipped with flame-ionization, with optimal elution conditions, as previously described in the mentioned study. Diets and muscle FA levels were calculated using the tricosanoid acid (C23:0) as internal standard and expressed as 100 g DM^−1^ or g 100 g^−1^ muscle wet weight (WW), respectively.

#### 2.6.5. Primary Metabolite Profiling Analysis

Primary metabolites were extracted from 50 mg dry weight of dorsal muscle samples from two fish per tank (six replicates per dietary treatment). Primary metabolites were extracted and derivatized as described by Lisec et al. [46]. Subsequently, primary metabolites were identified with gas chromatography time-of-flight mass spectrometry (GC-TOF-MS), as reported by Ferreira et al. [47]. The relative abundance of metabolite levels was normalized to the internal standard (ribitol) and the dry weight of the samples. Subsequently, fold-changes were determined and Log10-transformed for heatmap plotting.

### 2.7. Anterior Intestine Histomorphology

After fixation of the anterior intestine, cross-sections of three fish per tank (nine fish per dietary treatment) were selected for histological processing according to standard procedures. Tissues were embedded in paraffin and cut into cross-sectional sections of 4 μm by a semiautomated rotary microtome (Leica RM 2245, Leica Biosystems, Nussloch, Germany). For quantitative analysis of morphometric traits, a cross-section from each fish (nine slides per treatment) was stained with Alcian blue/Periodic Acid Schiff at pH 2.5 and digitized in high-resolution images. The cross-sectional perimeter, the muscularis thickness, the submucosa width, the lamina propria width, the absorption area, and the villus length were measured in each cross-section, and neutral (magenta) and acid (blue) goblet cells (GCs) were counted, following the methodology outlined by Ferreira et al. [48].

### 2.8. Calculations

The calculations for the assessment of growth performance and zootechnical parameters were carried out as described in Costa et al. [49]. Growth performance trajectories (e.g., weight gain and daily growth index) were estimated using mean body weight per tank at the beginning and end of the trial. The index of protein productive value (PPV) was estimated as follows: PPV (%) = Protein retention/protein intake × 100.

### 2.9. Statistical Analysis

Prior to analysis, data distributions were assessed for normality using either the Kolmogorov–Smirnov or Shapiro–Wilk tests, and homogeneity of variances was evaluated with Levene's test. When necessary, data transformations were applied to meet statistical assumptions. A one-way analysis of variance (ANOVA) was conducted using SPSS software (IBM SPSS Statistics 28, IL, USA), and treatment effects were further explored through Tukey's post hoc pairwise comparisons when significant differences were identified. For datasets that violated ANOVA assumptions, nonparametric analysis was performed using the Kruskal–Wallis test, followed by multiple comparisons of mean ranks to determine significant group differences. Metabolomic data were analyzed in R Studio [50] utilizing the “agricolae” [51], “gplots” [52], and “mixOmics” [53] packages. Treatment effects were examined through one-way ANOVA, consistent with the approach used for other datasets. Additionally, a supervised partial least squares discriminant analysis (PLS-DA) was carried out using leave-one-out cross-validation, as implemented in the “mixOmics” package. Across all analyses, results were considered statistically significant at a threshold of *p* < 0.05.

## 3. Results

### 3.1. Diets Characterization

All experimental diets remained isolipidic, isonitrogenous, and isoenergetic (Table 2) with a similar AA profile. The highest P, Ca, and Fe contents were observed in the FISH diet, while the lowest contents of P, Al, Ca, Fe, Co, and Cu were observed in the CTRL diet. All diets had equivalent FA profiles (Table 3), although the CTRL diet had the highest percentage of linoleic acid (C18:2n6).

### 3.2. Growth Performance and Whole-Body Composition

All diets were very well accepted by European seabass, resulting in similar VFI (1.4–1.5 g/100g ABW day) among dietary treatments. After 89 days, all groups had at least quadrupled their IBWs, resulting in a DGI of 1.6 (Table 4). Fish fed the SWINE diet presented a trend toward higher FBW (*p*=0.09) and had a significantly higher K compared to all other groups. The FCR was low (1.0–1.1) and did not differ significantly among dietary treatments.

Regarding somatic indexes, fish fed the SWINE diet had significantly higher HSI values compared to SHARK and CTRL groups, and higher VSI values compared to the CTRL group. The levels of macronutrients and energy in fish whole-body remained similar among dietary treatments. However, an increase in whole-body ash and P content was observed in the SHARK group.

### 3.3. Apparent Digestibility of Experimental Diets and Nutrient Balance

The digestibility of protein and lipids was high (> 95%) and did not differ among diets, as indicated in Table 5. No significant differences were found in the N balance parameters among dietary treatments. The energy apparent digestibility coefficient (ADC) of the FISH diet (93.1%) was significantly higher than that of the CTRL (91.5%) and SWINE diets (92.1%), which exhibited significantly higher fecal energy losses. FISH diet simultaneously had the highest digestible energy (DE) intake and metabolizable energy, but also had the highest total heat production and highest energy losses, resulting in a trend for an overall lower energy retention efficiency (% DE), but without statistical significance (*p*=0.05). The FISH diet exhibited significantly higher Fe ADC values (26%) compared to the SHARK and SWINE diets (19%–20%), which were themselves significantly higher than the CTRL diet, which had the lowest Fe digestibility (8%). Co ADC was significantly higher in the SHARK diet when compared to the CTRL and SWINE diets.

### 3.4. Muscle Proximal Composition and FA Profile

Regarding the muscle composition (Table 6), the SHARK group presented a trend (*p*=0.08) to contain lower protein than FISH and SWINE groups. Total lipids remained similar among groups, although some FAs present slight differences between dietary treatments. In all groups, monounsaturated FA (MUFA) was the most abundant FA class (42%–43%), followed by polyunsaturated FA (PUFA; 33%–34%) and saturated FA (SFA; 21%–22%). Palmitoleic acid (C16:1 n7) was present in significantly higher levels in the SHARK group compared to the FISH group. Fish fed SWINE diet had a significantly lower Σω6 level (12.5%) than the CTRL (13.4%) and FISH (13.1%) groups, resulting in the highest Σω3/Σω6 ratio (1.6 vs. 1.5). Furthermore, the muscle levels of eicosanoid acid (EPA) and docosahexaenoic acid (DHA) reached 0.5 g per 100 g of muscle WW in all dietary treatments.

### 3.5. Muscle Primary Metabolite Profile

**Figure 1:**
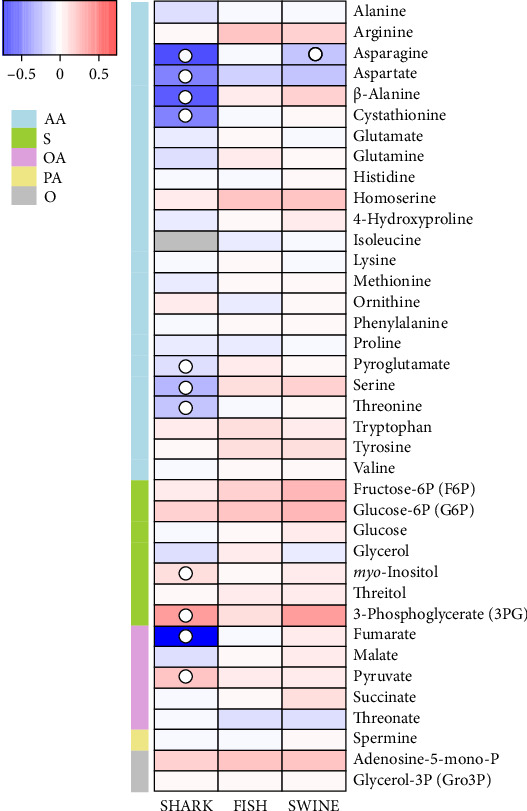
Heatmap visualization representing the changes in the relative levels of 38 primary metabolites in fish muscle tissue fed the experimental diets. Fold changes were calculated in relation to the CTRL dietary treatment. Significant changes (*p* < 0.05) are indicated as ○. Metabolites grouped in amino acids and derivatives (AA), sugars and derivatives (sugar phosphate; sugar alcohol; sugar acid; S), organic acids (OAs), polyamines (PAs), and others (O).

GC-TOF-MS analysis allowed the identification of 38 primary metabolites in European seabass dorsal muscle: namely, 23 AAs, seven sugars and derivatives, six organic acids, one polyamine, and two other primary metabolites, as shown in Tables S1 and S2 and Figure 1. The SHARK-fed fish group evidenced a significant decrease in asparagine, aspartate, β-alanine, cystathionine, pyroglutamate, serine, threonine, and fumarate contents and a significant increase in 3-phosphoglycerate, pyruvate, and *myo*-inositol contents when compared to CTRL-fed fish, as shown in Figure 1. Besides, isoleucine could not be identified in SHARK group. In the SWINE group, a significant reduction in asparagine was also identified compared to the CTRL group, while the FISH group maintained similar metabolite levels to the CTRL group.

**Figure 2:**
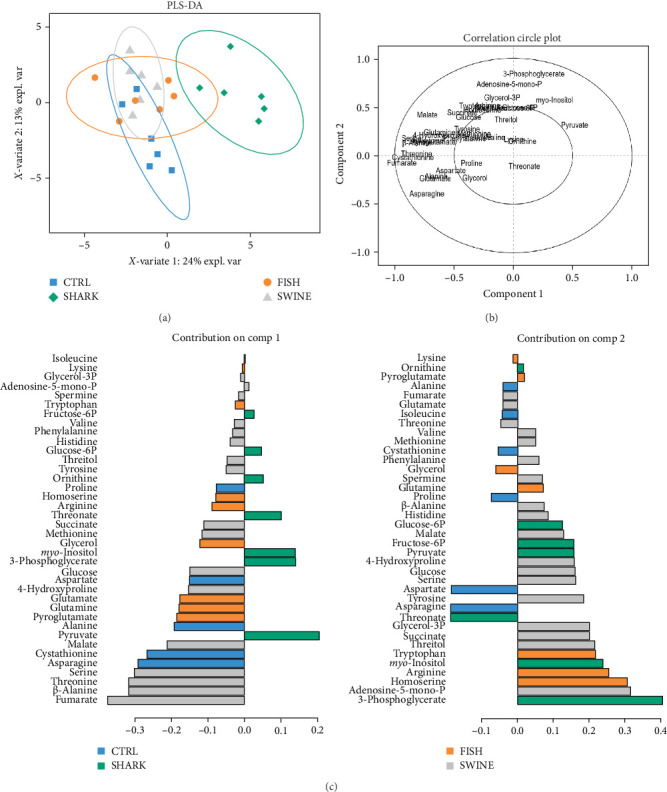
Partial least square discriminant analysis (PLS-DA) score plot (A) and correlation plot (B) of the primary metabolite profile in European seabass muscle tissues fed by the experimental diets. (C) PLS-DA contribution plots relative to component 1 and component 2 of the primary metabolite profile in fish muscle from all groups.

The supervised PLS-DA score plot of the first two components (principal component [PC] 1 and PC2) showed CTRL and SWINE groups clustered together and clearly separated from the SHARK group, particularly by the PC1, which explained 24 % of the total variance, as shown in Figure 2A. The FISH group cluster mostly overlapped the CTRL and SWINE clusters. The contribution plots (Figure 2C) showed that pyruvate, 3-phosphoglycerate, and *myo*-inositol were the metabolites that mostly contributed for cluster separation in PC1, and the same was for threonate, asparagine, and aspartate, which contributed cluster separation in PC2.

The circle plot revealed a high number of high correlated metabolites on the negative side of *x*-axis, which were responsible for the cluster of CTRL, FISH, and SWINE groups (Figure 2B). On the positive part of the *x*-axis, 3-phosphoglycerate, *myo*-inositol, and pyruvate, whose levels are significantly increased in SHARK dietary treatment, were responsible for the separation of SHARK group cluster.

### 3.6. Anterior Intestine Histomorphology

**Figure 3:**
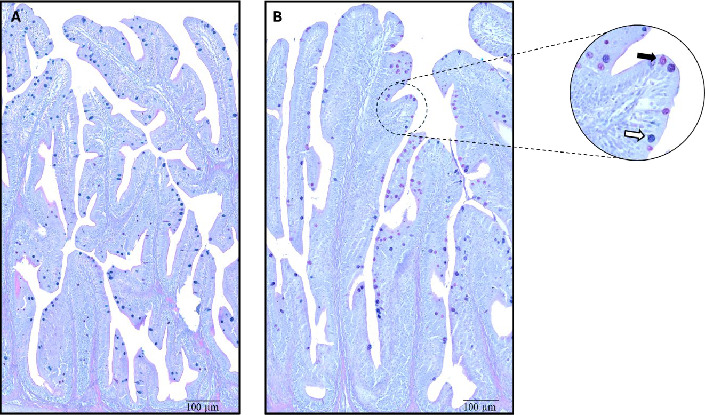
Goblet cells (GC) in transverse sections of anterior intestine (stained with AB/PAS) of European seabass fed with the following dietary treatments: (A) CTRL; (B) FISH. The arrows stand by colors: White—acid GC; Black—neutral GG.

The morphology of the anterior intestine was well preserved in all fish, with no disruption of structural integrity. Overall, the selected morphometric parameters did not vary significantly among dietary treatments, except the number of neutral GCs, which was significantly higher in the FISH group compared to the CTRL (Table 7 and Figure 3).

## 4. Discussion

The dietary inclusion of 3% locally-sourced animal protein hydrolysates enabled a reduction in FM inclusion from 12.5% to 8.5% without compromising feed intake, growth performance, feed efficiency, nutrient utilization, and muscle nutritional value, while preserving anterior gut integrity. The SWINE diet was associated with the highest FBW value and enhanced K. The FISH diet increased energy digestibility and the abundance of neutral GC in the anterior gut of fish. The SHARK diet led to higher whole-body ash and P content, with the muscle metabolome indicating a shift toward muscle protein catabolism to supply glucogenic AAs for energy, without compromising growth performance. All hydrolysate-supplemented diets improved Fe digestibility and preserved EPA + DHA levels in muscle tissue.

The reduction of high-quality FM inclusion by incorporating animal protein hydrolysates into highly plant-based diets for European seabass resulted in comparable feed intake, growth performance, and feed efficiency to the CTRL diet. Previous studies have shown that reducing FM levels to levels below 10% in nutritionally balanced diets for European seabass juveniles compromised growth and protein efficiency after 60 days [30]. Castro et al. [31] further demonstrated that lowering FM inclusion to less than 10% in plant-based diets over a long-term trial resulted in lower FBW, specific growth rate, and feed efficiency. Similarly, Qian et al. [27] highlighted that low FM levels in plant-based diets for carnivorous species can suppress VFI, ultimately hindering fish growth performance. In contrast, the present study showed that FM inclusion could be further reduced to 8.5% by incorporating 3% locally-sourced animal protein hydrolysates, without compromising feed intake, growth performance, or feed efficiency. Numerous studies [19, 54, 55] have suggested that hydrolysates may enhance feed attractiveness and help mitigate the reduced palatability often associated with plant-based diets [7, 56]. Gisbert et al. [57] showed that including 5% shrimp protein hydrolysate in plant-based diets for European seabass enabled a reduction of FM from 20% to 5% without affecting growth performance. However, unlike the present study, their lower FM diet required increased dietary supplementation with L-lysine and L-methionine. This underscores the potential of locally-sourced animal protein hydrolysates in diets with minimal FM inclusion, eliminating the need for additional AA supplementation or other additives to support growth and feed efficiency.

Overall, nutrient utilization was similar across dietary treatments, resulting in comparable whole-body composition. High inclusion of PP often impairs protein ADC in several fish species [58–61], including European seabass [62], but this could not be confirmed in the present study. The high and similar protein ADC values (≃96 %) across dietary treatments suggest that the dietary proteins were digested and utilized in a similar manner, resulting in comparable N gain. Resende et al. [21] demonstrated that incorporating swine blood hydrolysate, processed using different methods, into plant-based diets for European seabass resulted in varying effects on protein digestibility, emphasizing the importance of both the hydrolysate source and processing method. Although there is no statistical significance in the ADC of lipids between dietary treatments, the trend observed for the SHARK and FISH diets, which showed the highest values (*p*=0.09), may explain the increased energy ADC observed in these same dietary treatments. The reduction of FM inclusion associated with high plant-based diets are often problematic for carnivorous species, due to the presence of high levels of ANFs and indigestible fibers in plant ingredients, which are often responsible for enteritis and reduced feed utilization [63–68]. However, this could not be observed in the present study as gut integrity and absorption capacity remained similar across groups. Leduc et al. [8] found that reducing dietary FM inclusion from 20% to 5% in a plant-based diet for European seabass led to a decrease in villus length and GC number in the anterior gut. However, supplementing this diet with a 5% mixture of FISH_Hs restored these parameters to levels comparable to the higher FM diet. These results align with the present observations, which showed that intestinal morphology remained unaffected by the diets. Although the inclusion of FISH_H resulted in an increased number of neutral GCs in sea bass, total GCs number and GCs area remained similar among treatments. GCs are responsible for the production of mucins (glycosylated proteins), which are the primary components of the mucus layer in the fish intestine [69]. Different types of mucins perform multiple functions, including mucosa protection against pathogen infections and digestive enzymes, mucosa surface lubrification, microbiota modulation, or acting as a nutrient diffusion barrier [70]. Based on the presence or absence of acid groups in the mucin structure, they are classified as acid or neutral mucins, respectively [71]. In our study, FISH diet appears to differentially modulate the abundance of neutral mucins in GC present in the anterior gut of European seabass. Although previous studies have hypothesized that neutral mucins (secreted by neutral GC) could play a role in digestion and absorption processes in fish [72–74], no improvement in protein ADC was observed in the FISH group. While no major alterations were detected in the anterior intestinal section, the potential for diet-induced immune responses in the posterior intestine remains to be investigated.

The inclusion of the hydrolysates in feed formulation enhanced Fe bioavailability for European seabass. In teleosts, Fe uptake occurs via the divalent metal transporter 1 (DMT-1) present in the anterior gut epithelium [75]. However, when Fe forms soluble peptide–Fe complexes, it may follow alternative absorption routes mediated by peptide transporters [76]. Although the peptide sequences of the tested hydrolysates were not characterized, the enhanced Fe ADC observed in fish fed diets containing hydrolysates could be attributed to the potential presence of bioactive peptides with chelating properties present in these ingredients. However, this effect appears to be selective for Fe, as no consistent improvements were observed for other minerals tested. According to Cai et al. [76], the selectivity for specific metals relies on the compatibility between peptide–metal binding affinity (determined by peptide sequences) and the intrinsic chemical properties of each ion. Moreover, the higher presence of electron-rich side chain AAs with Fe-chelating properties (e.g., asparagine + aspartic acid, glutamic acid + glutamine, and cystine) [77] found in FISH_H [35], may have contributed to the further improvement in Fe ADC in this treatment compared to the all other diets. Nevertheless, further studies are needed to clarify the chelating mechanisms and gut transporters involved. Since plant ingredients often contain ANFs that reduce mineral availability [78], identifying ingredients with counteracting effects is crucial to ensuring that the mineral requirements of fish are met.

Fish fed the SWINE group showed a trend toward increased FBW (*p*=0.09), while the K and somatic indexes were significantly higher in fish fed this diet, suggesting increased lipid deposition in the liver and viscera due to diet-induced metabolic changes. Similarly, Teoh and Wong [79] reported that including 2% shrimp hydrolysate in a plant-based diet for catfish (*Pangasius hypophthalmus*) increased HSI and VSI. In contrast, Xu et al. [80] showed that including 10% and 20% of FISH_Hs in turbot plant-based diets reduced both somatic indexes. Therefore, it seems that the inclusion of hydrolysates from different sources and at varying doses in feed can modulate European seabass lipid metabolism in distinct ways. In the present study, despite the observed increases in somatic indices, whole-body protein and lipid contents remained consistent across dietary treatments. Moreover, although the tested hydrolysates had a lower fat content (<0.3%) compared to FM (7%), the nutritional quality of the fillets from European seabass fed the corresponding diets remained similar. No differences were observed in the EPA + DHA content amongst groups, all containing ~0.5 g per 100 g fresh weight, the maximum recommended value by the European Food Safety Authority (EFSA; 0.25–0.50 g/day) for preventing cardiovascular disease in healthy adults (EFSA Panel on Dietetic Products and Allergies, 2010) [81].

The muscle metabolic profile was significantly impacted by diets including hydrolysates, particularly in fish fed the SHARK diet. This effect was mainly due to decreased levels of several free AAs and fumarate, an intermediate of the tricarboxylic acid (TCA) cycle, together with an increase in glycolytic metabolites, such as 3-phosphoglycerate and pyruvate. Such changes may indicate alterations in AA metabolism in muscle, possibly reflecting adjustments in energy metabolism [82]. In addition, possible differences in protein turnover rates may provide a complementary explanation, as they could further affect the pool of free AAs [83]. The higher accumulation of 3-phosphoglycerate (glycolytic intermediate) and pyruvate (end-product of glycolysis) may further indicate that glycolytic activity exceeded the capacity of pyruvate dehydrogenase to incorporate pyruvate into TCA cycle [84]. In parallel, the increase in *myo*-inositol levels, a molecule involved in the regulatory mechanisms of muscle glucose transporters translocation [85–88], may also suggest a compensatory adjustment. Although all diets tested had a similar and balanced AA profile, the lower contribution of EAAs from SHARK_H, together with its reduced protein solubility, when compared to other hydrolysates [41], may have limited the rapid postabsorptive availability of these AAs for muscle uptake, possibly contributing to the compensatory metabolic adjustments observed in this group. Wei et al. [17] reported that replacing FM with ≥5.4% ultrafiltrated FISH_H in plant-based diets for turbot reduced muscle levels of fumarate and glucogenic AAs, while growth performance was only impaired at the 10.8% inclusion level. The authors suggested that these metabolic alterations indicate the fish had reached their tolerance limit to hydrolysate inclusion (or FM replacement). Exceeding this level ultimately led to metabolic changes that translated into reduced fish growth. Although different hydrolysate inclusion levels were not tested in this study, it is noteworthy that the metabolic alterations observed in European seabass fed the SHARK diet resemble those reported at 5.4% dietary hydrolysate inclusion in the referenced study. Although no differences in growth performance or nutrient retention were observed between the CTRL and SHARK groups, the tested inclusion level may already approach a threshold at which metabolic adjustments in muscle become evident. These findings suggest that, in terms of muscle metabolic regulation, SHARK_H may not be as effective as other hydrolysates in replacing FM. This underscores the need for future studies evaluating different SHARK_H inclusion levels to determine its optimal use in European seabass diets. However, despite these metabolic differences, no significant effects were observed on muscle protein deposition or growth performance, as all groups quadrupled their initial body weight.

## 5. Conclusion

The inclusion of agri-food by-product hydrolysates in plant-based diets effectively reduced FM inclusion to 8.5%, ensuring high feed intake, growth performance, feed efficiency, nutrient utilization, gut integrity, and muscle nutritional value. All diets supplemented with hydrolysates enhanced Fe digestibility in European seabass, suggesting the beneficial mineral chelation capacity of these hydrolysates. The inclusion of SWINE_H in the diet appears to induce metabolic modulation, resulting in higher somatic indexes. On the other hand, the disruption in the muscle metabolic profile of fish fed by SHARK diet suggests that the lower EAAs content in SHARK_H may compromise muscle basal metabolism, increasing reliance on tissue protein for energy. This metabolomic approach suggests that prolonged use of such hydrolysate in European seabass diets could impair muscle growth in the long-term. Further studies are needed to clarify the impact of hydrolysates on the muscle protein turn-over process. Upcycling meat and fish by-products into protein hydrolysates with bioactive potential supports zero-waste practices within the circular bioeconomy and provides locally-sourced alternatives to replace limited FM for aquafeeds.

## Figures and Tables

**Table 1:** Ingredients of the experimental diets.

Diets	CTRL	SHARK	FISH	SWINE
Ingredients (%)				
Fishmeal LT70^a^	12.5	8.5	8.9	8.5
SHARK_H^b^	—	3.0	—	—
FISH_H^c^	—	—	3.0	—
SWINE_H^d^	—	—	—	3.0
Soy protein concentrate^e^	25.0	25.0	25.0	25.0
Wheat gluten^f^	14.4	14.4	14.4	14.4
Corn gluten meal^g^	10.0	10.0	10.0	10.0
Soybean meal^h^	11.0	11.0	11.0	11.0
Wheat meal^i^	8.9	9.2	8.9	9.2
Vitamin and mineral premix^j^	1.0	1.0	1.0	1.0
Choline chloride 50% SiO_2_	0.2	0.2	0.2	0.2
Antioxidant^k^	0.2	0.2	0.2	0.2
Monocalcium phosphate	2.5	2.9	2.9	2.9
Yttrium oxide	0.02	0.02	0.02	0.02
Fish oil^l^	7.4	7.8	7.8	7.8
Rapeseed oil^m^	6.8	6.8	6.8	6.8

^a^Fishmeal NORVIK LT. Sopropêche, France (72% CP, 7% CF).

^b^Hydrolysate from blue shark (*Prionace glauca*) skin (Total fraction)—enzymatic hydrolysis with alcalase.

^c^Hydrolysate: enzymatic hydrolysis with alcalase.

^d^Hydrolysate from swine by-products (mono-species)—pressure and heat hydrolysis.

^e^Soy protein concentrate Soycomil-P, ADM, Animal Nutrition, Netherlands (62% CP, 0.7% CF).

^f^Wheat gluten, Roquette Frères, France (80% CP, 7% CF).

^g^Corn gluten meal COPAM, Portugal (61% CP, 6% CF).

^h^Dehulled solvent extracted soybean meal, Cargill, Spain (48% CP, 2% CF).

^i^Wheat meal Casa Lanchinha Lda., Portugal (11% CP, 2% CF).

^j^Vitamin and mineral premix WISIUM MIX AQUA 1.5%, ADM Portugal S.A., Portugal.

^k^Antioxidant powder (Verdilox): VERDILOX, Kemin Europe NV, Belgium.

^l^Sardine oil, Sopropêche, France.

^m^Rapeseed oil, Henry Lamotte Oils GmbH, Germany.

**Table 2:** Proximate composition, amino acid profile, and mineral composition of the experimental diets.

Diets	CTRL	SHARK	FISH	SWINE
Proximate composition (g 100 g^−1^ DM or kJ/g DM)				
Dry matter (DM, g 100 g^−1^)	95.3	94.5	92.8	95.1
Ash	6.6	7.2	7.2	7.2
Crude protein	52.5	53.2	52.7	53.1
Crude fat	17.8	17.7	18.2	17.6
Energy (kJ/g DM)	22.4	22.9	23.0	22.8
Amino acid profile (g 100 g^−1^ DM)				
Essential amino acids (EAAs)				
Arginine	3.0	3.0	2.9	3.0
Histidine	1.2	1.1	1.1	1.1
Lysine	2.6	2.6	2.6	2.6
Threonine	2.0	1.9	2.0	1.9
Isoleucine	2.4	2.4	2.4	2.3
Leucine	4.2	3.9	4.2	4.0
Valine	2.4	2.3	2.4	2.3
Methionine	1.3	1.3	1.3	1.3
Phenylalanine	2.9	2.7	2.7	2.8
ΣEAA	21.9	21.3	21.6	21.4
Nonessential amino acids (NEAA)				
Cystine	1.1	1.0	1.0	1.0
Tyrosine	2.3	2.3	2.3	2.3
Aspartic acid + asparagine	4.4	4.4	4.4	4.3
Glutamic acid + glutamine	10.8	9.8	10.0	9.8
Alanine	2.5	2.6	2.6	2.6
Glycine	2.5	2.8	2.5	2.7
Proline	3.7	3.4	3.4	3.5
Serine	2.7	2.5	2.6	2.5
ΣNEAA	29.9	28.7	28.7	28.7
NEAA/EAA	1.37	1.35	1.33	1.34
ΣAA	51.9	50.0	50.3	50.2
Mineral composition (µg g^−1^ DM)				
Aluminum	119	131	132	131
Calcium	10,314	11,325	11,894	11,749
Manganese	106	105	118	125
Iron	174	187	200	192
Cobalt	0.2	0.3	0.3	0.3
Copper	23	24	24	24
Phosphorus	9664	10359	10603	10367
Yttrium	186	190	193	194

**Table 3:** Fatty acid profile of the experimental diets.

Diets	CTRL	SHARK	FISH	SWINE
Fatty acids (g 100 g^−1^total fatty acids)				
C14:0	3.2	3.3	3.2	3.2
C16:0	11.7	11.7	11.5	11.6
C18:0	2.4	2.4	2.4	2.4
ΣSFA^a^	18.8	18.7	18.5	18.6
C16:1 n7 (PA)	3.8	3.9	3.8	3.8
C18:1 n9 (OA)	33.3	33.5	34.2	34.1
C20:1 n9	1.2	1.2	1.3	1.3
C24:1 n9	0.3	0.3	0.3	0.3
ΣMUFA^b^	42.5	42.7	43.5	43.4
C18:2 n6 (LA)	15.1	14.6	14.5	14.5
C18:3 n3 (ALA)	3.5	3.6	3.6	3.6
C18:4 n3	1.2	1.2	1.1	1.2
C20:4 n6	0.4	0.4	0.4	0.4
C20:3 n3	0.03	0.03	0.03	0.03
C20:5 n3 (EPA)	7.2	7.4	7.1	7.2
C22:5 n3	0.7	0.7	0.7	0.7
C22:6 n3 (DHA)	5.5	5.5	5.4	5.4
EPA + DHA	12.7	12.8	12.5	12.6
ΣPUFA^c^	35.9	35.8	35.2	35.2
ΣPUFA n-3	18.4	18.7	18.3	18.4
ΣPUFA n-6	15.7	15.2	15.2	15.1

Abbreviations: ALA, α-linolenic acid; DHA, docosahexaenoic; DM, dry matter; EPA, eicosapentaenoic acid; LA, linoleic acid; MUFA, monounsaturated fatty acid; OA, oleic acid; PA, palmitoleic acid; PUFA, polyunsaturated fatty acid; SFA, saturated fatty acid.

^a^Includes C10:0, C12:0, C15:0, C17:0, C20:0, C22:0, and C24:0.

^b^Includes: C14:1, C15:1, C17:1n-7, C18:1n-7, C22:1n-11, and C22:1n-9.

^c^Includes C16:2n-4, C16:3n-4, C16:4n-1, C18:3n-6, C20:2n-6, C20:3n-6, and C20:4n-3.

**Table 4:** Growth performance, feed utilization, somatic indices, and final whole-body composition of European seabass fed the experimental diets for 89 days.

Diets	CTRL	SHARK	FISH	SWINE	*p*-Value
Growth performance					
Final body weight (FBW) (g)	52.1 ± 8.3	53.0 ± 10.2	52.9 ± 9.3	54.8 ± 9.1	0.09
Final body length (FBL) (cm)	16.3 ± 0.8	16.5 ± 1.0	16.4 ± 0.9	16.4 ± 0.8	0.60
Weight gain (g)	39.4 ± 2.4	40.4 ± 3.3	40.2 ± 2.4	41.9 ± 4.4	0.81
Final condition factor (K)	1.20 ± 0.1^b^	1.19 ± 0.1^b^	1.19 ± 0.1^b^	1.22 ± 0.1^a^	0.002
Daily growth index (DGI)	1.6 ± 0.1	1.6 ± 0.1	1.6 ± 0.1	1.6 ± 0.1	0.83
Voluntary feed intake (VFI) (g/100 g ABW day)	1.4 ± 0.04	1.5 ± 0.1	1.5 ± 0.1	1.5 ± 0.03	0.18
Feed conversion ratio (FCR)	1.0 ± 0.01	1.1 ± 0.004	1.1 ± 0.03	1.1 ± 0.03	0.20
Protein efficiency ratio (PER)	1.8 ± 0.02	1.8 ± 0.01	1.8 ± 0.1	1.8 ± 0.1	0.11
Protein productive value index (PPV) (%)	31.7 ± 1.7	30.8 ± 0.3	30.8 ± 0.9	30.2 ± 0.8	0.46
Mortality (%)	0.7 ± 1.2	2.0 ± 2.0	1.3 ± 1.2	1.3 ± 1.2	0.73
Somatic indexes (%)					
Hepatosomatic index (HSI)	1.4 ± 0.3^b^	1.4 ± 0.2^b^	1.5 ± 0.4^ab^	1.6 ± 0.3^a^	0.004
Viscerosomatic index (VSI)	7.2 ± 1.1^b^	7.7 ± 1.4^ab^	7.7 ± 1.5^ab^	8.4 ± 1.5^a^	0.02
Whole body composition (%WW)					
Dry matter	36.6 ± 0.6	36.4 ± 0.4	35.9 ± 1.1	36.4 ± 0.7	0.70
Ash	3.2 ± 0.2^b^	3.8 ± 0.2^a^	3.3 ± 0.1^b^	3.4 ± 0.2^ab^	0.01
Protein	17.1 ± 0.6	17.1 ± 0.2	17.3 ± 0.1	17.1 ± 0.4	0.90
Lipids	14.9 ± 0.9	15.0 ± 0.2	14.7 ± 1.2	15.0 ± 1.3	0.99
Energy (kJ g^−1^)	10.2 ± 0.3	9.6 ± 0.3	9.6 ± 0.4	9.8 ± 0.5	0.32
Phosphorus	0.43 ± 0.03^b^	0.54 ± 0.05^a^	0.47 ± 0.003^ab^	0.48 ± 0.02^ab^	0.02

*Note:* Values represent mean ± standard deviation (*n* = 3 except for FBW, FBL, and K, where *n* = 150, and HSI and VSI, where *n* = 30). Initial whole-body composition (% or kJ/g wet weight): DM, 30.2; ash, 4.4; protein, 16.4; lipids, 9.4; energy, 67.4; phosphorus, 0.7. In each row, different letters indicate significant differences between treatments (*p* < 0.05).

Abbreviations: ABW, average body weight; WW, wet weight.

**Table 5:** Apparent digestibility coefficients (ADCs) and nutrient balances of the experimental diets.

Diets	CTRL	SHARK	FISH	SWINE	*p*-Value
ADC (%)					
Dry matter	77.8 ± 0.5	79.4 ± 0.4	78.1 ± 0.5	78.6 ± 1.1	0.09
Protein	95.9 ± 0.2	96.1 ± 0.1	96.0 ± 0.24	96.0 ± 0.1	0.81
Lipids	98.2 ± 0.04	98.5 ± 0.02	98.5 ± 0.3	98.4 ± 0.2	0.09
Energy	91.5 ± 0.4^c^	92.6 ± 0.1^ab^	93.1 ± 0.1^a^	92.1 ± 0.5^bc^	0.003
Calcium	27.9 ± 4.5	35.3 ± 1.4	34.7 ± 2.5	36.3 ± 4.2	0.06
Manganese	30.7 ± 5.6	31.5 ± 2.7	37.7 ± 2.9	39.4 ± 5.5	0.10
Iron	8.2 ± 1.6^c^	19.4 ± 1.6^b^	25.8 ± 1.4^a^	19.9 ± 2.3^b^	<0.001
Cobalt	30.9 ± 1.0^b^	46.4 ± 4.5^a^	40.8 ± 2.2^ab^	35.4 ± 6.6^b^	0.01
Copper	78.6 ± 3.4	81.5 ± 1.0	83.2 ± 0.6	80.7 ± 3.0	0.18
Phosphorus	67.1 ± 0.9	67.2 ± 2.5	68.0 ± 3.4	66.9 ± 2.3	0.95
Nitrogen (N) balance (g/kg ABW/day)					
Digestible N intake	1.1 ± 0.03	1.2 ± 0.04	1.2 ± 0.04	1.2 ± 0.02	0.11
N gain	0.4 ± 0.02	0.4 ± 0.01	0.4 ± 0.01	0.4 ± 0.02	0.89
NRE (%DN)	33.0 ± 1.8	32.1 ± 0.3	32.1 ± 0.9	31.5 ± 0.8	0.45
Fecal N losses	0.05 ± 0.001	0.05 ± 0.002	0.05 ± 0.002	0.05 ± 0.001	0.30
Metabolic N losses	0.8 ± 0.03	0.8 ± 0.03	0.8 ± 0.04	0.8 ± 0.01	0.08
Total N losses	0.8 ± 0.04	0.9 ± 0.03	0.9 ± 0.04	0.9 ± 0.01	0.09
Energy (E) balance (kJ/kg ABW/day)					
Digestible E intake	292.3 ± 8.8^b^	309.2 ± 10.6^ab^	319.5 ± 11.8^a^	317.2 ± 6.4^ab^	0.03
E gain	151.7 ± 4.6	141.9 ± 6.1	143.1 ± 10.1	146.9 ± 12.9	0.57
ERE (% DE)	51.9 ± 1.6	45.9 ± 1.8	44.8 ± 2.6	46.3 ± 4.2	0.05
Metabolizable E	273.1 ± 8.2^b^	289.0 ± 9.9^ab^	299.0 ± 10.8^a^	296.3 ± 6.2^ab^	0.03
Fecal E losses	27.1 ± 0.8^a^	24.7 ± 0.8^b^	23.8 ± 0.9^b^	27.1 ± 0.5^a^	0.002
Branchial and urinary E losses	19.1 ± 0.8	20.2 ± 0.7	20.5 ± 1.0	21.0 ± 0.2	0.08
Total heat production	121.4 ± 7.6^b^	147.0 ± 8.8^ab^	156.0 ± 9.2^a^	149.4 ± 14.2^a^	0.02
Total E losses	167.7 ± 8.6^b^	192.0 ± 10.0^ab^	200.2 ± 10.4^a^	197.4 ± 14.6^a^	0.03
Phosphorus (P) balance (g/kg ABW/day)					
Digestible P intake	0.09 ± 0.003^b^	0.10 ± 0.003^a^	0.11 ± 0.004^a^	0.10 ± 0.002^a^	0.001
Phosphorus gain	0.05 ± 0.01^b^	0.07 ± 0.01^a^	0.06 ± 0.002^ab^	0.06 ± 0.004^ab^	0.02
PRE (% DP)	53.1 ± 6.6^b^	68.4 ± 8.7^a^	52.0 ± 2.0^b^	56.7 ± 2.8^ab^	0.03
Fecal P losses	0.045 ± 0.001^b^	0.050 ± 0.002^a^	0.051 ± 0.002^a^	0.052 ± 0.001^a^	0.004
Metabolic P losses	0.043 ± 0.005^ab^	0.032 ± 0.010^b^	0.052 ± 0.003^a^	0.045 ± 0.002^ab^	0.02
Total P losses	0.089 ± 0.005^ab^	0.082 ± 0.01^b^	0.102 ± 0.005^a^	0.097 ± 0.001^ab^	0.02

*Note:* Values represent mean ± standard deviation. In each row, different letters indicate significant differences between treatments (*p* < 0.05).

Abbreviations: ABW, average body weight; ADC, apparent digestibility coefficient; EI, energy intake; ERE, energy retention efficiency; NI, nitrogen intake; NRE, nitrogen retention efficiency; PI, phosphorus intake; PRE, phosphorus retention efficiency.

**Table 6:** Total lipids, protein, and fatty acids profile the dorsal muscle of European seabass fed the experimental diets for 89 days.

Diets	CTRL	SHARK	FISH	SWINE	*p*-Value
Muscle composition (%WW)					
Total lipids	3.5 ± 0.2	3.8 ± 0.7	3.8 ± 0.5	4.5 ± 0.2	0.26
Protein	19.5 ± 0.8	18.6 ± 0.5	20.4 ± 0.7	19.8 ± 0.9	0.08
Fatty acids (g 100 g^−1^ total fatty acids)					
C14:0	2.3 ± 0.03	2.3 ± 0.07	2.2 ± 0.05	2.2 ± 0.07	0.07
C16:0	14.6 ± 0.3	14.8 ± 0.3	14.2 ± 0.3	14.5 ± 0.4	0.13
C18:0	3.7 ± 0.1	3.6 ± 0.1	3.7 ± 0.1	3.8 ± 0.1	0.13
ΣSFA^1^	21.4 ± 0.4	21.5 ± 0.3	20.9 ± 0.4	21.2 ± 0.3	0.15
C16 :1 n7	3.4 ± 0.02^ab^	3.5 ± 0.13^a^	3.3 ± 0.04^b^	3.4 ± 0.07^ab^	0.02
C18:1 n9 (OA)	32.8 ± 0.1	33.8 ± 0.7	33.3 ± 0.7	34.3 ± 0.8	0.10
C20:1 n9	1.6 ± 0.08	1.6 ± 0.08	1.6 ± 0.07	1.5 ± 0.05	0.29
C24:1 n9	0.26 ± 0.004^ab^	0.24 ± 0.03^ab^	0.27 ± 0.01^a^	0.22 ± 0.01^b^	0.03
ΣMUFA^2^	41.7 ± 0.01	42.7 ± 0.7	42.2 ± 0.7	42.9 ± 1.0	0.23
C18:2 n6 (LA)	11.9 ± 0.1^a^	11.2 ± 0.3^bc^	11.7 ± 0.3^ab^	11.1 ± 0.1^c^	0.01
C18:3 n3 (ALA)	2.6 ± 0.02	2.6 ± 0.05	2.7 ± 0.06	2.6 ± 0.05	0.26
C18:4 n3	0.7 ± 0.01	0.7 ± 0.01	0.7 ± 0.01	0.7 ± 0.03	0.90
C20:4 n6	0.7 ± 0.1	0.6 ± 0.1	0.6 ± 0.05	0.6 ± 0.1	0.75
C20:3 n3	0.1 ± 0.008	0.04 ± 0.001	0.05 ± 0.003	0.05 ± 0.003	0.39
C20:5 n3 (EPA)	6.8 ± 0.2	6.8 ± 0.2	7.0 ± 0.1	6.9 ± 0.2	0.72
C22:5 n3	0.9 ± 0.01	0.8 ± 0.005	0.9 ± 0.03	0.9 ± 0.06	0.23
C22:6 n3 (DHA)	8.2 ± 0.2	8.0 ± 0.2	8.6 ± 0.2	8.1 ± 0.6	0.24
EPA + DHA	15.0 ± 0.3	14.8 ± 0.4	15.6 ± 0.3	15.0 ± 0.8	0.35
ΣPUFA^3^	33.7 ± 0.4	32.6 ± 0.4	34.0 ± 0.8	32.6 ± 0.7	0.05
Σω3	19.6 ± 0.4	19.3 ± 0.4	20.2 ± 0.4	19.5 ± 0.8	0.26
Σω6	13.4 ± 0.2^a^	12.6 ± 0.2^bc^	13.1 ± 0.3^ab^	12.5 ± 0.1^c^	0.003
Σω3/Σω6	1.5 ± 0.03	1.5 ± 0.05	1.5 ± 0.01	1.6 ± 0.07	0.11
Fatty acids (%WW)					
C20:5 n3 (EPA)	0.2 ± 0.02	0.2 ± 0.04	0.2 ± 0.02	0.2 ± 0.004	0.94
C22:6 n3 (DHA)	0.3 ± 0.02	0.3 ± 0.05	0.3 ± 0.03	0.3 ± 0.02	0.87
EPA + DHA	0.5 ± 0.04	0.5 ± 0.09	0.5 ± 0.05	0.5 ± 0.02	0.92

*Note:* Values are presented as mean ± standard deviation; *n* = 3 (3 pools of 5 muscle samples/dietary treatment). Different superscript letters represent significant differences (*p* < 0.05); one-way ANOVA (post hoc Tukey's test).

Abbreviations: ALA, α-linolenic acid; DHA, docosahexaenoic; EPA, eicosapentaenoic acid; LA, linoleic acid; MUFA, monounsaturated fatty acid; OA, oleic acid; PUFA, polyunsaturated fatty acid; SFA, saturated fatty acid; WW, wet weight.

^1^Includes C10:0. C12:0. C15:0. C17:0. C20:0. C22:0, and C24:0.

^2^Includes: C14:1. C15:1. C17:1n-7. C18:1n-7. C22:1n-11, and C22:1n-9.

^3^Includes C16:2n-4. C16:3n-4. C16:4n-1. C18:3n-6. C20:2n-6. C20:3n-6, and C20:4n-3.

**Table 7:** Anterior intestine morphology of European seabass fed the experimental diets for 89 days.

Diets	CTRL	SHARK	FISH	SWINE	*p*-Value	
Cross-sectional perimeter (mm)	14.9 ± 1.7	13.0 ± 1.9	14.1 ± 1.7	13.4 ± 1.9	0.15	
Absorption area (mm^2^)	6.2 ± 1.0	5.2 ± 1.4	6.6 ± 1.2	6.4 ± 1.8	0.16	
Muscularis thickness (μm)	106.6 ± 24.9	114.1 ± 31.3	90.2 ± 21.5	110.9 ± 25.9	0.47	
Submucosa width (μm)	33.0 ± 8.3	34.1 ± 7.9	32.5 ± 6.0	30.8 ± 3.7	0.77	
Lamina propria width (μm)	31.1 ± 5.9	26.6 ± 5.4	29.3 ± 9.1	27.8 ± 1.8	0.44	
Villi length (μm)	1345.1 ± 262.3	1270.9 ± 155.7	1460.2 ± 235.1	1463.1 ± 223.2	0.21	
Acid globlet cells	1190.3 ± 423.3	793.3 ± 510.5	900.6 ± 478.6	792.2 ± 337.7	0.09	
Neutral globlet cells	233.3 ± 174.6^b^	318.0 ± 277.2^ab^	518.6 ± 148.7^a^	482.1 ± 242.8^ab^	0.03	
Total globlet cells	1423.7 ± 546.9	1111.3 ± 501.7	1419.1 ± 408.5	1274.3 ± 436.4	0.47	
Average globlet cells area (μm^2^)	49.1 ± 6.4	46.9 ± 5.0	51.9 ± 8.4	46.0 ± 6.0	0.25	

*Note:* Values represent mean ± standard deviation (*n* = 9). In each row, different letters indicate significant differences between treatments (*p* < 0.05).

## Data Availability

The data supporting this study are available from the corresponding author upon reasonable request.

## Supporting Information

Supporting Information The supporting information includes Table S1. Fold changes in relative values of 38 primary metabolites found in European seabass in relation to CTRL treatment; Table S2. Metabolite levels expressed as relative abundances, normalized to sample weight and the internal standard (ribitol), for the 38 primary metabolites identified in European seabass muscle.
