# Supplementary material for: Locally-Sourced Animal Protein Hydrolysates in High-Plant-Protein Diets Can Promote European Seabass Growth and Nutrient Utilization, Reducing Reliance on Fishmeal
Source: Aquac Nutr. 2025 Nov 18;2025:3415083. doi: 10.1155/anu/3415083 (PMC12646735; doi:10.1155/anu/3415083)
Supplement: Supporting Information — The supporting information includes Table S1. Fold changes in relative values of 38 primary metabolites found in European seabass in relation to CTRL treatment; Table S2. Metabolite levels expressed as relative abundances, normalized to sample weight and the internal standard (ribitol), for the 38 primary metabolites identified in European seabass muscle. [file 3415083.f1.docx]

| **Table S1.** Fold changes in relative values of 38 primary metabolites found in European seabass in relation to CTRL treatment. | | | | | |
| --- | --- | --- | --- | --- | --- |
| **Metab Class** | **Analyte** | **CTRL** | **SHARK** | **FISH** | **SWINE** |
| **AA** | Alanine | 1.00 ± 0.07 | 0.80 ± 0.06 | 0.97 ± 0.09 | 0.99 ± 0.05 |
|  | Arginine | 1.00 ± 0.18 | 1.04 ± 0.13 | 1.49 ± 0.33 | 1.36 ± 0.13 |
|  | Asparagine | 1.00 ± 0.10 | **0.32 ± 0.08** | 0.97 ± 0.20 | **0.71 ± 0.05** |
|  | Aspartate | 1.00 ± 0.22 | **0.44 ± 0.10** | 0.77 ± 0.13 | 0.68 ± 0.20 |
|  | beta-Alanine | 1.00 ± 0.18 | **0.37 ± 0.04** | 1.10 ± 0.24 | 1.37 ± 0.17 |
|  | Cystathionine | 1.00 ± 0.08 | **0.46 ± 0.11** | 1.00 ± 0.22 | 1.01 ± 0.16 |
|  | Glutamate | 1.00 ± 0.03 | 0.85 ± 0.07 | 1.02 ± 0.07 | 0.98 ± 0.05 |
|  | Glutamine | 1.00 ± 0.09 | 0.80 ± 0.09 | 1.16 ± 0.15 | 1.08 ± 0.15 |
|  | Histidine | 1.00 ± 0.07 | 0.98 ± 0.06 | 0.98 ± 0.03 | 1.04 ± 0.06 |
|  | Homoserine | 1.00 ± 0.20 | 1.14 ± 0.13 | 1.51 ± 0.15 | 1.42 ± 0.15 |
|  | 4-Hydroxyproline | 1.00 ± 0.06 | 0.90 ± 0.11 | 1.04 ± 0.07 | 1.16 ± 0.10 |
|  | Isoleucine | 1.00 ± 0.06 | n.d. | 0.91 ± 0.18 | 1.00 ± 0.05 |
|  | Lysine | 1.00 ± 0.08 | 0.99 ± 0.05 | 1.04 ± 0.05 | 0.99 ± 0.11 |
|  | Methionine | 1.00 ± 0.12 | 0.87 ± 0.11 | 1.01 ± 0.11 | 1.04 ± 0.06 |
|  | Ornithine | 1.00 ± 0.17 | 1.11 ± 0.20 | 0.87 ± 0.13 | 1.08 ± 0.23 |
|  | Phenylalanine | 1.00 ± 0.03 | 0.98 ± 0.04 | 1.02 ± 0.12 | 1.02 ± 0.04 |
|  | Proline | 1.00 ± 0.10 | 0.86 ± 0.12 | 0.86 ± 0.06 | 0.97 ± 0.11 |
|  | Pyroglutamate | 1.00 ± 0.04 | **0.84 ± 0.05** | 1.11 ± 0.09 | 1.01 ± 0.10 |
|  | Serine | 1.00 ± 0.10 | **0.63 ± 0.06** | 1.28 ± 0.10 | 1.30 ± 0.11 |
|  | Threonine | 1.00 ± 0.04 | **0.71 ± 0.03** | 0.95 ± 0.05 | 1.04 ± 0.06 |
|  | Tryptophan | 1.00 ± 0.05 | 1.09 ± 0.09 | 1.19 ± 0.11 | 1.12 ± 0.07 |
|  | Tyrosine | 1.00 ± 0.06 | 1.04 ± 0.09 | 1.28 ± 0.25 | 1.26 ± 0.16 |
|  | Valine | 1.00 ± 0.06 | 0.99 ± 0.07 | 1.03 ± 0.12 | 1.03 ± 0.03 |
| **S** | Fructose-6P (F6P) | 1.00 ± 0.29 | 1.16 ± 0.17 | 1.33 ± 0.38 | 1.52 ± 0.49 |
|  | Glucose-6P (G6P) | 1.00 ± 0.21 | 1.38 ± 0.18 | 1.43 ± 0.42 | 1.56 ± 0.47 |
|  | Glucose | 1.00 ± 0.06 | 0.95 ± 0.06 | 1.05 ± 0.04 | 1.10 ± 0.05 |
|  | Glycerol | 1.00 ± 0.15 | 0.81 ± 0.07 | 1.09 ± 0.19 | 0.89 ± 0.03 |
|  | myo-Inositol | 1.00 ± 0.04 | **1.26 ± 0.08** | 1.08 ± 0.09 | 1.18 ± 0.09 |
|  | Threitol | 1.00 ± 0.12 | 1.03 ± 0.06 | 1.10 ± 0.11 | 1.18 ± 0.06 |
|  | 3-Phosphoglycerate (3PG) | 1.00 ± 0.20 | **1.90 ± 0.18** | 1.27 ± 0.09 | 1.85 ± 0.36 |
| **OA** | Fumarate | 1.00 ± 0.09 | **0.19 ± 0.02** | 0.99 ± 0.08 | 1.14 ± 0.07 |
|  | Malate | 1.00 ± 0.07 | 0.83 ± 0.09 | 1.05 ± 0.07 | 1.15 ± 0.08 |
|  | Pyruvate | 1.00 ± 0.10 | **1.47 ± 0.09** | 1.16 ± 0.11 | 1.09 ± 0.11 |
|  | Succinate | 1.00 ± 0.13 | 0.93 ± 0.08 | 1.09 ± 0.14 | 1.21 ± 0.09 |
|  | Threonate | 1.00 ± 0.14 | 0.97 ± 0.07 | 0.80 ± 0.07 | 0.78 ± 0.06 |
| **PA** | Spermine | 1.00 ± 0.09 | 0.99 ± 0.09 | 0.99 ± 0.10 | 1.08 ± 0.12 |
| **O** | Adenosine-5-mono-P | 1.00 ± 0.42 | 1.33 ± 0.27 | 1.46 ± 0.35 | 1.48 ± 0.22 |
|  | Glycerol-3P (Gro3P) | 1.00 ± 0.05 | 1.03 ± 0.04 | 1.02 ± 0.03 | 1.08 ± 0.04 |
| Bold values indicate statistical differences *p* < 0.05 in relation to CTRL dietary treatment. The abbreviations stand for: AA – amino acid and derivates; S – sugars and derivates; OA – organic acids; PA – polyamines; O – others. n.d. not detected. | | | | | |

| **Table S2.** Metabolite levels expressed as relative abundances, normalized to sample weight and the internal standard (ribitol), for the 38 primary metabolites identified in European seabass muscle. | | | | | |
| --- | --- | --- | --- | --- | --- |
| **Metab Class** | **Analyte** | **CTRL** | **SHARK** | **FISH** | **SWINE** |
| **AA** | Alanine | 0.9 ± 0.2 | 0.7 ± 0.1 | 0.9 ± 0.2 | 0.9 ± 0.1 |
|  | Arginine | 0.002 ± 0.001 | 0.002 ± 0.001 | 0.003 ± 0.002 | 0.003 ± 0.001 |
|  | Asparagine | 0.08 ± 0.02 | 0.03 ± 0.02 | 0.08 ± 0.04 | 0.06 ± 0.01 |
|  | Aspartate | 0.06 ± 0.03 | 0.03 ± 0.02 | 0.05 ± 0.02 | 0.04 ± 0.03 |
|  | beta-Alanine | 0.01 ± 0.004 | 0.003 ± 0.001 | 0.01 ± 0.005 | 0.01 ± 0.003 |
|  | Cystathionine | 0.003 ± 0.001 | 0.001 ± 0.001 | 0.003 ± 0.002 | 0.003 ± 0.001 |
|  | Glutamate | 0.2 ± 0.01 | 0.1 ± 0.03 | 0.2 ± 0.03 | 0.2 ± 0.02 |
|  | Glutamine | 0.2 ± 0.04 | 0.2 ± 0.04 | 0.2 ± 0.08 | 0.2 ± 0.07 |
|  | Histidine | 0.8 ± 0.1 | 0.7 ± 0.1 | 0.7 ± 0.1 | 0.8 ± 0.1 |
|  | Homoserine | 0.002 ± 0.001 | 0.002 ± 0.001 | 0.003 ± 0.001 | 0.003 ± 0.001 |
|  | 4-Hydroxyproline | 0.3 ± 0.05 | 0.3 ± 0.1 | 0.3 ± 0.04 | 0.3 ± 0.06 |
|  | Isoleucine | 0.03 ± 0.004 | n.d. | 0.03 ± 0.01 | 0.03 ± 0.003 |
|  | Lysine | 0.1 ± 0.02 | 0.1 ± 0.01 | 0.1 ± 0.01 | 0.1 ± 0.03 |
|  | Methionine | 0.02 ± 0.01 | 0.02 ± 0.01 | 0.02 ± 0.01 | 0.02 ± 0.003 |
|  | Phenylalanine | 0.02 ± 0.001 | 0.02 ± 0.002 | 0.02 ± 0.01 | 0.02 ± 0.002 |
|  | Proline | 0.9 ± 0.2 | 0.8 ± 0.3 | 0.8 ± 0.1 | 0.9 ± 0.2 |
|  | Pyroglutamate | 0.34 ± 0.03 | 0.29 ± 0.04 | 0.38 ± 0.1 | 0.34 ± 0.1 |
|  | Serine | 0.2 ± 0.05 | 0.1 ± 0.03 | 0.2 ± 0.05 | 0.2 ± 0.05 |
|  | Threonine | 0.15 ± 0.01 | 0.11 ± 0.01 | 0.14 ± 0.02 | 0.16 ± 0.02 |
|  | Tryptophan | 0.01 ± 0.002 | 0.01 ± 0.003 | 0.02 ± 0.004 | 0.01 ± 0.002 |
|  | Tyrosine | 0.02 ± 0.003 | 0.02 ± 0.005 | 0.03 ± 0.01 | 0.03 ± 0.01 |
|  | Valine | 0.07 ± 0.01 | 0.1 ± 0.01 | 0.08 ± 0.02 | 0.08 ± 0.01 |
| **S** | Fructose-6P (F6P) | 0.01 ± 0.005 | 0.01 ± 0.003 | 0.01 ± 0.01 | 0.01 ± 0.01 |
|  | Glucose-6P (G6P) | 0.001 ± 0.001 | 0.002 ± 0.001 | 0.002 ± 0.001 | 0.002 ± 0.001 |
|  | Glucose | 0.1 ± 0.02 | 0.1 ± 0.02 | 0.1 ± 0.01 | 0.2 ± 0.02 |
|  | Glycerol | 0.04 ± 0.01 | 0.03 ± 0.01 | 0.04 ± 0.02 | 0.03 ± 0.002 |
|  | myo-Inositol | 0.03 ± 0.003 | 0.03 ± 0.01 | 0.03 ± 0.01 | 0.03 ± 0.01 |
|  | Threitol | 0.01 ± 0.002 | 0.01 ± 0.001 | 0.01 ± 0.002 | 0.01 ± 0.001 |
|  | 3-Phosphoglycerate (3PG) | 0.001 ± 0.001 | 0.002 ± 0.001 | 0.002 ± 0.0003 | 0.002 ± 0.001 |
| **AO** | Fumarate | 0.01 ± 0.003 | 0.002 ± 0.001 | 0.01 ± 0.002 | 0.01 ± 0.002 |
|  | Malate | 0.03 ± 0.005 | 0.0 ± 0.01 | 0.03 ± 0.005 | 0.03 ± 0.005 |
|  | Pyruvate | 0.05 ± 0.01 | 0.1 ± 0.01 | 0.05 ± 0.01 | 0.05 ± 0.01 |
|  | Succinate | 0.02 ± 0.01 | 0.02 ± 0.004 | 0.02 ± 0.01 | 0.03 ± 0.004 |
|  | Threonate | 0.001 ± 0.0005 | 0.001 ± 0.0003 | 0.001 ± 0.0002 | 0.001 ± 0.0002 |
|  | Ornithine | 0.1 ± 0.04 | 0.1 ± 0.04 | 0.1 ± 0.03 | 0.1 ± 0.04 |
| **PA** | Spermine | 0.001 ± 0.0003 | 0.001 ± 0.0002 | 0.001 ± 0.0003 | 0.001 ± 0.0003 |
| **O** | Adenosine-5-mono-P | 0.03 ± 0.04 | 0.05 ± 0.02 | 0.05 ± 0.03 | 0.05 ± 0.02 |
|  | Glycerol-3P (Gro3P) | 0.1 ± 0.01 | 0.1 ± 0.0 | 0.1 ± 0.01 | 0.1 ± 0.01 |
| The abbreviations stand for: AA – amino acid and derivates; S – sugars and derivates; OA – organic acids; PA – polyamines; O – others. n.d. not detected. | | | | | |
